# Letter to Veugelers, P.J. and Ekwaru, J.P., A Statistical Error in the Estimation of the Recommended Dietary Allowance for Vitamin D. *Nutrients* 2014, *6*, 4472–4475; doi:10.3390/nu6104472

**DOI:** 10.3390/nu7031688

**Published:** 2015-03-10

**Authors:** Robert Heaney, Cedric Garland, Carole Baggerly, Christine French, Edward Gorham

**Affiliations:** 1 Creighton University, Omaha, NE 68178, USA; 2 Department of Family and Preventive Medicine, University of California, San Diego, La Jolla, CA 92093, USA; E-Mails: cgarland@ucsd.edu (C.G.); Edward.Gorham@med.navy.mil (E.G.); 3 GrassrootsHealth, Encinitas, CA 92024, USA; E-Mails: carole@grassrootshealth.org (C.B.); french.cmb@gmail.com (C.F.)

Recently Veugelers and Ekwaru published data [1] indicating that, in its dietary reference intakes for calcium and vitamin D, the Institute of Medicine (IOM) had made a serious calculation error [2]. Using the same data set as had the IOM panel, these investigators showed that the Recommended Dietary Allowance (RDA) for vitamin D had been underestimated by an order of magnitude. Veugelers and Ekwaru, using the IOM’s data, calculated an RDA of 8895 IU per day. They noted that there was some uncertainty in that estimate, inasmuch as this value required an extrapolation from the available data, which did not include individuals receiving daily vitamin D inputs above 2400 IU/day.

In this communication, we present data from a different cohort entirely, including many individuals with vitamin D intakes spanning a range from zero to above 10,000 IU per day. The data presented here are derived from the GrassrootsHealth (GRH) database, which had been characterized elsewhere [3]. Here we examine the probability range for the previously published GRH data at all supplement intakes across the relevant range.

**Figure 1:**
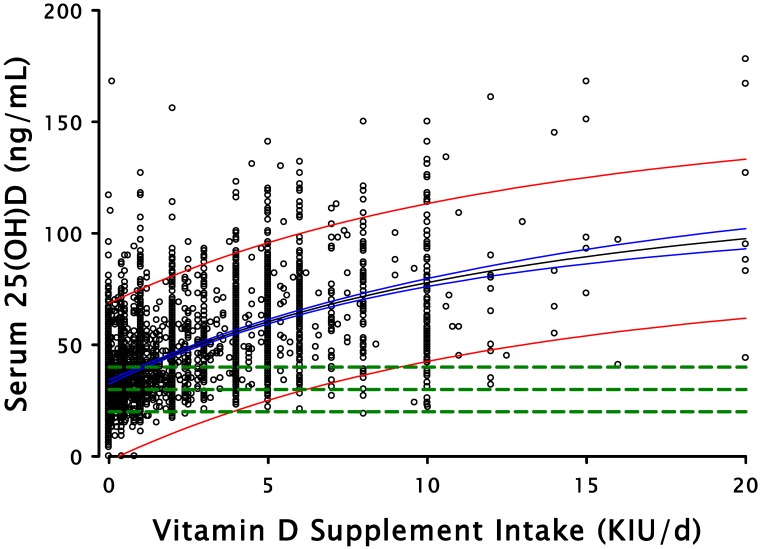
Serum 25(OH)D plotted against vitamin D supplement intake.

Figure 1 plots the 25(OH)D values for 3657 individuals as a function of their own intakes of vitamin D, showing both the best fit regression line through the data [3] and the 95% confidence limits for the total data set. The horizontal dashed lines in the figure are for serum 25(OH)D values of 20, 30, and 40 ng/mL (50, 75, and 100 nmol/L). The points at which these lines intersect the lower bound of the 95% probability band for serum 25(OH)D reflect the inputs necessary to ensure that 97.5% of the cohort would have a vitamin D status value at or above the respective 25(OH)D concentration. These three values represent, respectively, the recommended values of the IOM, the Endocrine Society [4], and GrassrootsHealth. The precise inputs corresponding to these serum 25(OH)D values are 3875, 6201, and 9122 IU/day.

While the 3875 IU intake value needed to achieve at least 20 ng/mL (50 nmol/L) in 97.5% of the population is lower than the estimate of Veugelers and Ekwaru, it should be noted first that, as Veugelers and Ekwaru had stated, both estimates are roughly an order of magnitude higher than the published IOM value. Also, it must be stressed that this input is explicitly supplemental, i.e., it presumes a daily, basal input from food and sun of some non-zero magnitude. The best-fit regression line through the data, as can be seen in the figure, intersects the *Y*-axis at a value of 34 ng/mL (85 nmol/L), reflecting an intake from food and sun amounting to somewhat more than 3000 IU per day (5). Since an RDA, by definition, relates to intake from all sources, it is clear that total intake required to achieve 20 ng/mL in 97.5% of the cohort must be close to 7000 IU per day, not substantially different from that calculated by Veugelers and Ekwaru.

Thus, we confirm the findings of these investigators with regard to the published RDA for vitamin and we call for the IOM and all public health authorities concerned with transmitting accurate nutritional information to the public to designate, as the RDA, a value of approximately 7000 IU per day from all sources. We note that this conclusion applies specifically to the IOM’s designation of 20 ng/mL as the lower bound of adequacy, and that higher values, such as that of the Endocrine Society and GRH, would mandate the higher RDA values cited above.

With regard to possible safety concerns related to such a recommendation, we note that: (a) as the figure shows, the mean 25(OH)D and the upper bound of the 95% probability range for the supplemental intake of 3875 IU/day are less than 50 ng/mL and 100 ng/mL, respectively; (b) the correctly calculated RDA is well below the cutaneous production of vitamin D from summer sun [5]; and (c) the total, *all-source* intake of 7000 IU/day is below the no observed adverse effect level (NOAEL) of both the IOM and the Endocrine Society, below the tolerable upper intake level (UL) of the Endocrine Society, and well within the safe range delineated by Hathcock *et al.* [6], who had generated that range using the IOM’s method of hazard identification.
